# Cirrhosis Classification Based on Texture Classification of Random Features

**DOI:** 10.1155/2014/536308

**Published:** 2014-02-24

**Authors:** Hui Liu, Ying Shao, Dongmei Guo, Yuanjie Zheng, Zuowei Zhao, Tianshuang Qiu

**Affiliations:** ^1^Department of Biomedical Engineer, Dalian University of Technology, Dalian 116024, China; ^2^Department of Radiology, Second Affiliated Hospital, Dalian Medical University, Dalian 116027, China; ^3^Department of Radiology, University of Pennsylvania, Philadelphia, PA 19104, USA; ^4^Second Affiliated Hospital, Dalian Medical University, Dalian 116027, China

## Abstract

Accurate staging of hepatic cirrhosis is important in investigating the cause and slowing down the effects of cirrhosis. Computer-aided diagnosis (CAD) can provide doctors with an alternative second opinion and assist them to make a specific treatment with accurate cirrhosis stage. MRI has many advantages, including high resolution for soft tissue, no radiation, and multiparameters imaging modalities. So in this paper, multisequences MRIs, including T1-weighted, T2-weighted, arterial, portal venous, and equilibrium phase, are applied. However, CAD does not meet the clinical needs of cirrhosis and few researchers are concerned with it at present. Cirrhosis is characterized by the presence of widespread fibrosis and regenerative nodules in the hepatic, leading to different texture patterns of different stages. So, extracting texture feature is the primary task. Compared with typical gray level cooccurrence matrix (GLCM) features, texture classification from random features provides an effective way, and we adopt it and propose CCTCRF for triple classification (normal, early, and middle and advanced stage). CCTCRF does not need strong assumptions except the sparse character of image, contains sufficient texture information, includes concise and effective process, and makes case decision with high accuracy. Experimental results also illustrate the satisfying performance and they are also compared with typical NN with GLCM.

## 1. Introduction

Liver cirrhosis is one of the leading causes of death by disease [1]. Making a definite diagnosis and staging of the cirrhosis is crucial which will help doctor to offer the timely and appropriate therapeutic method. In recent decades, CAD has drawn an increasing attention for its convenient and noninvasive diagnosis procedure, meaningful diagnosis result. As it is well known, the more cirrhosis categories, the more valuable the classification result is, for it can provide a constructive assistance to doctors and facilitate the process for doctors to produce a specific treatment for patient. Therefore, the study of cirrhosis classification is developing form two categories classification of cirrhosis, such as methods provided by Chen et al. [2], Lee et al. [1, 3], Hui et al. [4], and Li et al. [5], to precisely class three stages (normal stage, early stage, and middle and advanced stage). Thus, a CAD system which can generate more precise stages is an inevitable tendency in the future.

The early CAD systems of cirrhosis mostly apply CT images, such as the methods provided by Li et al. [5] and Chen et al. [2, 6]. Compared with CT, MRI provides many advantages including high resolution of soft tissue, no radiation, and multiparameters imaging modalities and it is becoming an effective modality in assessing cirrhosis. However, few researchers are concerned with the cirrhosis CAD system with MRI by far. So in this paper, multisequences MRI, including T1-weighted, T2-weighted, arterial phase, portal venous phase, and equilibrium phase, are applied for cirrhosis classification which divide cases into three stages (normal stage, early stage, and middle and advanced stage).

Cirrhosis CAD usually consists of two steps: the first step involves feature extraction and effective feature selection, and the second step is to train classifier based on the selected features and identify cirrhosis [4]. Feature extraction and selection occupy an important position that directly affects the performance of classifier. Cirrhosis is characterized by the presence of widespread fibrosis and regenerative nodules in the hepatic. The fibrosis and nodules formation causes distortion of the normal hepatic architecture, resulting in characteristic texture patterns [1]. Therefore, extracting texture feature is the primary task of CAD. Jiang et al.'s cirrhosis classification method [7] achieves 56% accuracy with texture features directly extracted from region of interests (ROI), such as the ROI mean gray value and ROI standard deviation. Because simple texture features are unsatisfactory, Jiang et al. add morphological features to improve classification performance. Actually, effective texture feature that can fully describe cirrhosis feature is a key problem of CAD. GLCM based feature is a classical method to extract texture features for cirrhosis classification [4, 8–11]. However, GLCM has many weaknesses. For example, GLCM is a statistical texture description for two specific grayscales with specific distance and direction which will cause strong assumptions about the texture being studied in practice. And it is difficult to contain all texture information according to the GLCM texture features with such strong assumptions. It should also be noted that complex process of computing GLCM and its 14 typical texture parameters will take long time especially for large image. And with GLCM based features it will need a necessary procedure to select effective features with a heavily and complicated test to improve the classifier's performance. Virmani et al. [9] uses all the 14 GLCM texture features for two stages classification (normal and cirrhosis). Its classification accuracy is 95.86%, achieved by using an NN classifier with stratified 10-fold cross validation method. For two stages classification, GLCM texture features are fine even though with the price of time and complexity. However, the classification with the GLCM texture features is far from satisfactory for three stages (normal, early, and middle and advanced); the accuracy is about 89.52% for normal, 70.98% for early, and 74.27% for middle and advanced stages that is verified in this paper, which causes most of traditional classifiers far from application.

Recently, texture classification from random features [12] proposed by Liu et al. provides an effective way to extract texture features. Firstly, it does not need strong assumptions about the texture images, except the sparse character of the texture image. Secondly, it almost does not lose information when extracting texture feature. Because no information is lost in the three stages which are extracting local image from image, extracting patch from local image, and stretching patch into patch vector, the process of compressing patch vector retains salient information when image is sparse character. Thus, the texture feature, which is compressed patch vector, almost does not lose information. Thirdly, Liu et al.'s method does not need feature selection, reducing method complexity. Finally, it costs little time because of the simple computation procedure which includes extracting local image from image, extracting patch from local image, stretching patch into patch vector, and compressing patch vector that only needs matrix multiplication. And omitting feature selection can also save time.

Considering the above reasons, we propose CCTCRF method and apply Liu et al.'s method to extract texture features. Liver organization of hepatic MRIs studied in this paper is a kind of image with texture character, and it also meets the sparse character requirement as an assumption in Liu et al.'s method. As it is well known, the key factor to obtain excellent performance in cirrhosis classification is preserving salient cirrhosis texture information, while Liu et al.'s method exactly meets the requirement according to the process of extracting texture feature. Furthermore, due to the weak assumption of Liu et al.'s method, we can apply it into five sequences MRIs all have sparse character without considering specific parameters for specific sequence, and the experiment result also demonstrates the effectiveness of the five sequences MRIs texture features. In addition, as the number of multisequences MRI used in our research is large, the little time costing and complexity reducing in feature extraction and selection is also a reason to choose Liu et al.'s method. Thus, we apply Liu et al.'s method as texture extraction method in our research. Except for the benefits of texture feature extraction based on Liu et al.'s method, CCTCRF also has a remarkable performance on case classification, compared with typical GLCM texture based CAD system.

In this paper, we propose the CCTCRF method with five sequences MRI, involving T1-weighted, T2-weighted, hepatic arterial phase, portal venous phase, and equilibrium phase images to class the patient samples into normal stage, early stage, and middle and advanced stage. In order to illustrate the effectiveness of CCTCRF method, we also do a contrast experiment with GLCM texture features and typical neural network (NN).

This paper is organized as follows.  Section 2 presents the materials used in our study and describes the NN classification method with GLCM texture features to be compared and the CCTCRF theory applied in this paper.  Section 3 describes the experiments performed to prove the effectiveness of CCTCRF and the NN classifier with GLCM texture features experiment as comparison study. The conclusion is in Section 4.

## 2. Materials and Methods

### 2.1. Materials

The MR images are collected from the Second Affiliated Hospital of Dalian Medical University between September 2012 and September 2013. The database containing 55 patient cases is used in this study. Of the 55 patient cases, 26 cases are at normal stage, 13 are early cirrhosis stage, and 16 are the middle and advanced cirrhosis, shown in Table 1.

Most of the 55 patient cases have five sequences MRI, T1-weighted, T2-weighted, arterial phase, portal venous phase, and equilibrium phase, except a few of them who are short of portal venous phase or equilibrium phase images. The cases are scanned by a 3.0-T superconductivity MR scanner (Signa, Siemens, Germany) or 1.5-T MR scanner (Signa, GE, USA); the scan order is T1-wighted, T2-wighted, and three kinds of dynamic enhancement scan which are arterial phase, portal venous phase, and equilibrium phase, respectively, after injecting Gd-DTPA 25 s, 65 s, and 120 s. The parameters of T1-weighted, arterial phase, portal venous phase, and equilibrium phase from 3.0-T superconductivity MR scanner are TR = 3.9 ms and TE = 1.4 ms; T2-weighted parameters are TE = 105 ms. The parameters of T1-weighted, arterial phase, portal venous phase, and equilibrium phase from 1.5-T MR scanner are TR = 175.0 ms and TE = 4.2 ms; T2-weighted parameters are TR = 7058.8 ms and TE = 89.5 ms.

### 2.2. Methods

#### 2.2.1. Texture Classification from Random Features

The method of texture classification from random features [12] is inspired by theories of sparse representation and compressed sensing. And it presents a simple and powerful approach for texture image classification based on random projection. It includes four parts, patch extraction, compressed texton dictionary, histogram of textons learning, and the classification. At the feature extraction step, texture feature, compressed patch vector, is extracted with patch extraction, patch vector generation, and patch vector compressing. The texture features are embedded into a bag-of-words model to perform texture classification. The extraction of compressed patch vector with random projection is simple and takes full advantages of the sparse nature of texture images. Liu et al.'s method outperforms traditional feature extraction methods which involve careful design and complex steps. Compared with the four state-of-the-art texture classification involving Patch, Patch-MRF, MR8, and LBP on the databases of CUReT, Brodatz, and MSRC, Liu et al.'s method leads to an important improvement in classification accuracy and reductions in feature dimensionality [12].

#### 2.2.2. NN Classification with GLCM Texture Features

NN classifier [13] is a classic method in pattern classification and recognition, widely used in speech recognition [14, 15], image recognition [16–18], handwritten character recognition [19, 20], radar target recognition [21], and sonar target recognition [22, 23]. NN simulates the progress of neure that obtains knowledge from outside world and learns by itself to achieve the class function. GLCM is a classic method to extract texture feature, provided by Haralick [24]. It describes the probability that a couple of pixels, at *θ* direction and at a distance of *d* pixels, appear *i* gray level and *j* gray level. Then, it deduces 14 texture features including angular second moment, contrast, correlation, variance, inverse differences moment, sum average, sum variance, sum entropy, entropy, difference variance, difference entropy, information measures of correlation, Maximum correlation coefficient 1, and Maximum correlation coefficient 2 [25] according to GLCM [26]. Thus, NN classifier with GLCM texture features is widely used in classification.

#### 2.2.3. Cirrhosis Classification Based on Texture Classification from Random Features (CCTCRF)

We propose a new method CCTCRF which distinguishes hepatic cirrhosis patients into normal stage, early stage, and middle and advanced stage with five sequences MRI, which are T1-weighted, T2-weighted, arterial phase, portal venous phase, and equilibrium phase. Liu et al.'s method is applied to extract texture features and class intermediate samples. In addition, a final case decision making is produced with the intermediate samples classification results. CCTCRF includes four parts: ROI segment, texture feature extraction, ROI classification system, and case decision.

**Figure 1:**
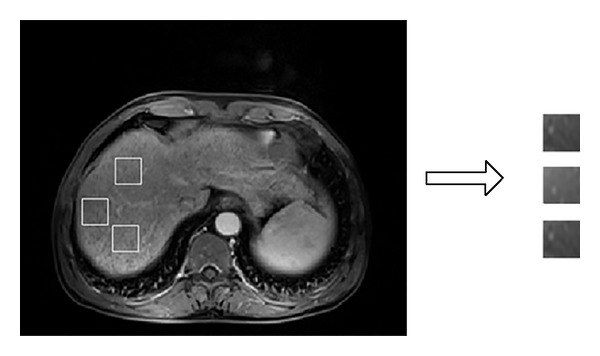
Extracting ROI.

ROI segment step is shown in Figure 1. The images used for segment are manually selected according to the requirements that the images need to contain a clear and relatively whole liver. ROIs with size of *n* × *n* are extracted from five sequences MRIs, and considering the extraction principle of the diffuse distribution of liver, large blood vessels within the liver are excluded [8]. In this paper, the size of ROI is 30 × 30 or 60 × 60, and it depends on the range in liver that can be extracted. And we extract many ROIs from the same image when it has enough region accord with the extraction principle.

**Figure 2:**
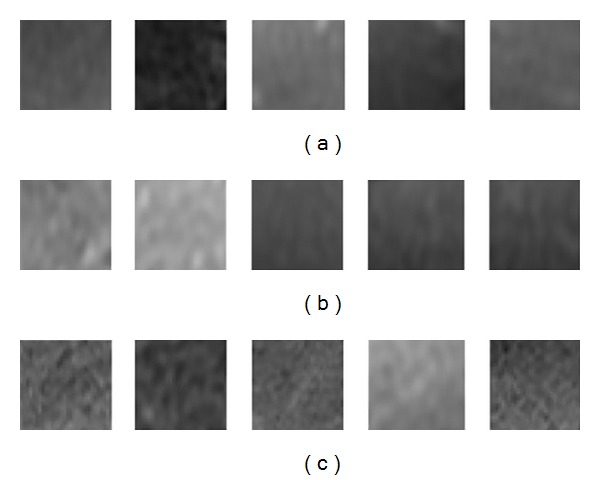
Normal, early, and middle and advanced stage ROIs form T1-weighted, T2-weighted, arterial phase, portal venous phase, and equilibrium phase.

Figures 2(a)–2(c) are the ROI examples of normal stage, early stage, and middle and advanced stage, respectively, and the image order is T1-weighted, T2-weighted, arterial phase, portal venous phase, and equilibrium phase for each stage. According to clinical information, hepatic MRI conveys the different texture information according to different cirrhosis stage: normal hepatic tissue appears as delicate texture and uniform medium gray level, while early cirrhosis and middle and advanced cirrhosis appear as coarse particles or diffuse small nodular with saltatory gray with different extent [4].

ROIs are manually segmented by an experienced radiologist; the number distribution of selected ROI is illustrated in Table 2.

At the feature extraction step, we extract texture feature from ROI. Processing a ROI of *n* ^2^ pixels by extracting square patch {*P*_*i*,*j*_} of *m* pixels and $\sqrt{m}$ is much less than *n*, from every pixel located in *P*_*i*,*j*_. And in order to get the square, those pixels on the ROI boundary are removed. So the range of *i*, *j* is $\left(\sqrt{m}/2,n-\sqrt{m}/2\right)$. Then, stretch each patch {*P*_*i*,*j*_} into a vector of size *m* and labelled as *p*_*l*_. Next, compress patch vector *p*_*l*_ with Φ, which is sampled from independent zero-mean, unit-variance normal distribution, to obtain compressed patch vector *x*_*l*_ = Φ*p*_*l*_ as the texture feature. Consider

(1) $$\begin{array}{c} X=\left\{x_{l}=\Phi p_{l}\;|\;p_{l}\in P\right\}, \end{array}$$

where **P** is the set of the patch generated from a ROI.

Before processing *x*_*l*_ as the input vector of classification, it needs to be normalized and there are two kind of normalization methods [12], formulated as (2) and (3) or no normalization as follows.

(1) Weber's law
  (2) $$\begin{array}{c} x⟵x\times \left[\frac{\log\left(1+{||x||}_{2}/0.03\right)}{{||x||}_{2}}\right]. \end{array}$$
(2) Unit norm
  (3) $$\begin{array}{c} x⟵\frac{x}{{||x||}_{2}}. \end{array}$$

Any kind of normalization can be selected based on the classification performance after the next process.

Patch vector, which comes from ROI, is the gray level assemble in practice. Thus, it reflects the different texture information of tissue and organ with T1 value and T2 value or proton density as well as gray level for MRI. It also reflects local information of a ROI and we can control the local range by adjusting *m* to achieve the ideal scope of cirrhosis texture. Actually, to compress patch vector is a process of dimensionality reduction and is important in handling high dimensional data since it mitigates the curse of dimensionality and other undesired properties of high dimensional spaces [12], and the compressed patch vector does not need to feature selection which has heavy and complicated algorithm or analysis. In this paper, random projection which refers to the technique of projecting a set of points from a high dimensional space to a randomly chosen low dimensional subspace is the way of compressing patch. Random projection's effectiveness in information-preserving and dimensionality-reduction power is evidenced in the emerging theory of compressed sensing (CS) [27–29], which states that, for sparse and compressible signals, a small number of nonadaptive linear measurements in the form of random projections can capture most of the salient information in the signal and allow for perfect reconstruction of the signal. And it has been widely used in information retrieval, face recognition [30], and machine learning [31, 32]. Thus, using the compressed patch vector *x* as the following input is reasonable and effective.

**Figure 3:**
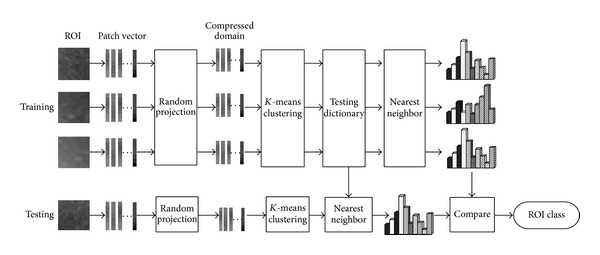
ROI training and testing.

**Figure 4:**
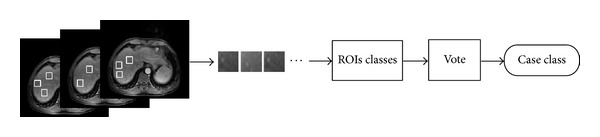
Case decision making step.

Our ROI classification system is illustrated in Figures 3-4, consisting of four steps. Suppose we have *C* cirrhosis stages and each stage has *S* samples.

(1)* Compressed Texton Dictionary Learning Step.* Compressed texton dictionary *W* is learned directly in the compressed domain **X**. It learns *K* textons with *K*-means for each stage compressed patch vectors. Next, the compressed texton dictionary *W* is formulated by concatenating the *K* textons of each texture stage and the size of dictionary is *CK* (i.e. *W* = *CK*).

(2)* Histogram of Textons Learning, Shown in Figure 3 (Top).* By labeling each of extracted patches *x* with the closest texton in *W*, *h*_*c*,*s*_ which is a histogram of compressed textons belonging to one of the samples from stage *c* is learned for each training sample. Each stage is represented by a set of models *H*_*c*_ = {*h*_*c*,*s*_}_*s*_ [12].

(3)* The Classification Step Is Shown in Figure 3 (Bottom).* Computing *h*_new_ which is a histogram from one of the samples belongs to test data. Using nearest neighbor classifier, *h*_new_ is classified and the distance between two histograms is measured using the *χ* ^2^ statistic:

(4) $$\begin{array}{c} \chi^{2}\left(h_{1},h_{2}\right)=\frac{1}{2}\overset{CK}{\underset{k=1}{\sum }}\frac{{\left[h_{1}\left(k\right)-h_{2}\left(k\right)\right]}^{2}}{h_{1}\left(k\right)+h_{2}\left(k\right)}. \end{array}$$

Then, *h*_new_ belongs to stage *i* when the distance between *h*_new_ and *h*_*i*_ is the nearest compared with other histograms. Thus, the stage of corresponding ROI which *h*_new_ derives from is determined; it is equal to *h*_new_ stage.

Case decision making stage is as follows. As we have shown in Figure 4, a case of one sequence images can extract many ROIs that the number of ROI is determined by the image region scope that meets extraction principle. In other words, *M* sequence images of case *A* have many classification results that belong to corresponding ROI. When all ROIs belong to the same case and same sequence is classed to the same stage *c*, surely the case of *M* sequence stage is *c*. However, when the ROIs classification results have more than one stage, case *A* of *M* sequence is determined by the vote principle that the minority stage subordinate to the majority stage.

## 3. Experiment Results

MATLAB R2010a is used to implement the CCTCRF experiment and the NN classifier with GLCM texture features experiment.

### 3.1. The CCTCRF Experiment Result

We use five sequences MRI in the experiment and the number of patients is shown in Table 1. Because images come from five different MR sequences, we need to do five experiments to class cases with the same sequence images in order to obtain the individual performance of every kind of sequence.

Take T1-weighted images for classification, for instance. There are 26 normal cases, 13 early cirrhosis cases, and 16 middle and advanced cirrhosis cases of T1-weighted. The train cases of normal stage, early stage, and middle and advanced stage are 13, 7, and 8; test cases are 13, 6, and 8. Extracting, respectively, 142, 93, and 235 ROIs from three kinds of cases according to the principle of extracting ROI and train cases of normal, early, and middle and advanced stage which are 54, 42, and 180 ROIs, respectively, the remaining ROIs belong to test cases. Each ROI is processed by extracting patches {*P*_*i*,*j*_} of size $\sqrt{25}\times \sqrt{25}$ around each pixel position (*i*, *j*) except those pixels on the ROI boundary. Stretching {*P*_*i*,*j*_} into *p*_*l*_, that is a vector of 25 pixels, we compressed *p*_*l*_ into a 15-dimension compressed patch vector using independent zero-mean, unit-variance normal distribution. Choose unit norm as normalization method on account of unit norm which has the best evaluation among the three normalization ways for T1-weighted. Then, the texture feature, compressed path vector with normalization, is achieved. Next, input the texture features into the ROI classification system, the distribution of cases number is shown in Table 3. After obtaining the ROIs stages, we carry out the case decision making step to achieve the test cases stages of T1-weighted.

What is said above is for T1-weighted images. Except the number of each stage case and parameters, T1-weighted, T2-weighted, arterial phase, portal venous phase, and equilibrium phase have the same process. The parameters in texture feature extraction stage include the size of ROI and patch and the dimension of compressed patch vector. In compressed texton dictionary learning step of ROI classification system, parameters include textons with *K*-means for each stage and which way to normalize compressed patch vector. We adjust these parameters according to the special sequence in order to achieve the desired performance of the sequence.

To evaluate the effectiveness of our study, we do five experiments using different sequences, respectively. The portal venous phase and equilibrium phase distribution are different from others because of the absence of portal venous phase in middle and advanced stage number 7, equilibrium phase in normal stage numbers 18 and 21–24, and middle and advanced stage numbers 6 and 7. Yet, we still keep the fact that the test cases are completely new compared with train cases.

The accuracy of every kind of sequence is demonstrated in Table 4. Firstly, it has a perfect result of case accuracy, that is, 100%, which means that all the cases can be classed correctly from case point. Secondly, ROI accuracy is not as good as case accuracy. Yet, it has completely no influence on case accuracy when taking all ROIs results of one case into account and it still has a remarkable performance, especially for normal stage. Normal stage can absolutely be separated from early stage and middle and advanced stage according to no matter ROI classification result or case classification result. To early stage, CCTCRF can almost be separated from others according to ROI classification result, except that ROI accuracy of T2-weighted images is 97.30%, because one of two ROIs of number 3 case is erroneous classified. Yet, case number 3 still classifies correctly after considering all the number 3 case ROIs.

Thirdly, the middle and advanced ROI accuracy is not as good as normal or early stage because the cirrhosis texture in middle and advanced liver is very irregular and is accompanied by morphological changes of liver which causes the difficulty to extract suitable ROI. However, it has no influence on case accuracy when considering all ROIs results, just like early stage number 3. In a word, all the cases have an encouraging case accuracy, that is, 100%, and maintain a remarkable ROI accuracy especially for normal and early stage which is more meaningful for doctors and patients.

### 3.2. NN Classifier with GLCM Texture Features in Comparison Study

**Figure 5:**
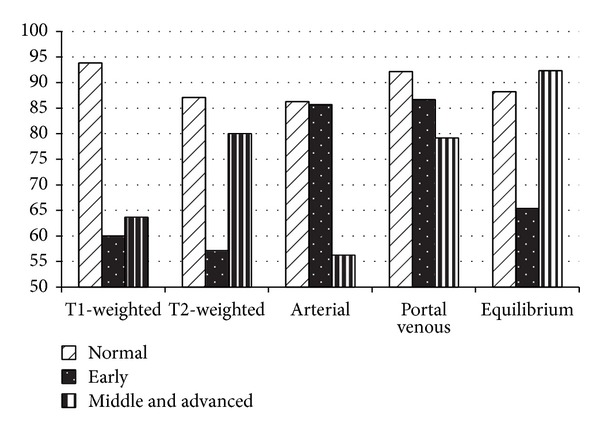
NN classification result.

We use five sequences MRI for NN classifier with GLCM texture features experiment and the number of patients is shown in Table 1. The texture features based on GLCM is classical statistics features. GLCM is a matrix that describes the probability of a couple of pixels whose gray levels are *i* and *j*, and the distance and direction between the couple pixels is *d* and *θ*. We did three stages (normal stage, early stage, and middle and advanced stage) classification experiment with 14 kinds of GLCM texture features of ROIs. And ROIs were extracted from MRI with the same extraction principle which is excluding the diffuse distribution of liver, large blood vessels within the liver [8]. We perform tenfold cross validation method to execute the classification and the result is shown in Figure 5. Obviously, the NN classifier with GLCM texture features does not have a harmonious or remarkable performance in cirrhosis classification with any sequence of MRI. However, it might be improved by adding feature selection and adjusting parameters, including adopting different angle and step to compute GLCM according to different MR sequence.

## 4. Conclusions

In this paper, we describe CCTCRF method with five sequences MRI, T1-weighted, T2-weighted, hepatic arterial phase, portal venous phase, and equilibrium phase, to class cirrhosis into three stages (normal, early, and middle and advanced stage). The experiment results show that CCTCRF method surpasses the typical classifier NN with GLCM texture features in cirrhosis classification, but with significant reductions in complexity and time. There are many advantages of CCTCRF than previous studies such as NN classifier with GLCM in cirrhosis texture classification.

Instead of GLCM texture features, we choose compressed patch vector as the texture features. Firstly, compared with GLCM which needs strong assumption including angle and step, compressed patch vector does not need strong assumptions about the texture images, except the sparse character of the image. And we apply the texture extraction method into five MRIs that all have sparse character without considering specific parameters adjusting for specific sequence and the experiment result also demonstrates the effectiveness of the five sequences MRI texture features. Secondly, it almost does not lose information when extracting texture feature, because no information is lost in the three steps which are extracting local image from image, extracting patch from local image, and stretching patch into patch vector. Meanwhile, the process of compressing patch vector process retains salient information when image is sparse. Thus, the texture feature, which is compressed patch vector, almost does not lose information. However, GLCM texture features must lose texture information under such a strong assumption condition. Thirdly, the texture features do not need feature selection, because it is already simplified with compressing and has a remarkable performance in experiment without feature selection. So, the omitting of feature selection reduces method complexity without reducing accuracy. Finally, CCTCRF texture feature extraction costs little time compared with GLCM texture feature extraction, which heavy computation burden and time-consuming are its big problem especially without feature selection. CCTCRF texture feature extraction has the simple texture feature extraction procedure which includes extracting local image from image, extracting patch from local image, stretching patch into patch vector, and compressing patch vector that only needs matrix multiplication. Meanwhile, omitting feature selection can also save time. Thus, the feature extraction of CCTCRF method is more suitable for the further popularization of cirrhosis CAD system that needs to provide diagnosis fast and accurately.

Further, CCTCRF experiment result has confirmed that the five sequences hepatic MRIs of T1-weighted, T2-weighted, arterial phase, portal venous phase, and equilibrium phase are all prominent for every stage cirrhosis classification. In addition, the case decision making step achieves a remarkable performance on the basis of ROI classification result which is already satisfying, and it eliminates few special ROIs inaccurate classification with an overall decision making of one case.

The promising results of this paper motivate a further research of cirrhosis diagnosis and classification. In the future, we will use more cases to verify CCTCRF's effectiveness and add morphological features into middle and advanced classification to improve its ROI accuracy. Furthermore, we will class the cirrhosis with more subtle stages with CCTCRF and apply CCTCRF in hepatic fibrosis classification which is more meaningful for doctors and patients and our medical development is our research direction in the future.

## Figures and Tables

**Table 1:** The number of collected cases.

	Normal	Early	Middle and advanced
T1-weighed	26	13	16
T2-weighed	26	13	16
Arterial	26	13	16
Portal venous	26	13	15
Equilibrium	20	13	14

**Table 2:** ROIs distribution.

	Normal	Early	Middle and advanced
T1-weighed	142	93	235
T2-weighed	75	64	91
Arterial	127	87	189
Portal venous	119	80	175
Equilibrium	94	84	153

**Table 3:** Distribution of cases numbers.

	Normal		Early		Middle and advanced	
	Test	Train	Test	Train	Test	Train
T1-weighted	1–13	14–26	1–7	8–13	1–8	9–16
T2-weighted	1–13	14–26	1–7	8–13	1–8	9–16
Arterial	1–13	14–26	1–7	8–13	1–8	9–16
Portal venous	1–13	14–26	1–7	8–13	1–6, 8, 9	10–16
Equilibrium	1–13	14–17, 19, 20, 25	1–7	8–13	1–5, 8, 9	10–16

**Table 4:** CCTCRF experiment result (%).

	Normal		Early		Middle and advanced	
	ROI	Case	ROI	Case	ROI	Case
T1-weighted	100	100	100	100	94.45	100
T2-weighted	100	100	97.30	100	91.89	100
Arterial phase	100	100	100	100	100	100
Venous	100	100	100	100	86.96	100
Equilibrium	100	100	100	100	98.48	100
